# Case Report: Novel *CXCR2* compound heterozygous variants in an infant with neutropenia

**DOI:** 10.3389/fimmu.2026.1845797

**Published:** 2026-05-29

**Authors:** Hua Liu, Yinzhu Zhang, Xiao Liu, Jiayue Qin, Chunyi Zhang, Junjin Huang, Danyang Zhang, Hebing Zhou, Ming Yang, Yan Yue

**Affiliations:** 1 Department of Pediatrics, Beijing United Family Women’s and Children’s Hospital, Beijing, China; 2 Department of Medical Affairs, Acornmed Biotechnology Co., Ltd., Beijing, China; 3 Department of Hematology, Beijing Lu He Hospital, Beijing, China

**Keywords:** case report, compound heterozygous variants, congenital neutropenia, CXCR2, recurrent infections

## Abstract

Congenital neutropenia refers to a group of rare inherited disorders in which neutrophil development or release is disrupted, predisposing affected children to frequent infections. Among the genes involved, *CXCR2* loss-of-function variants have recently been recognized as a distinct cause of the disease. Here, we report a 12-month-old Chinese boy, who presented with repeated febrile respiratory illnesses and intermittent neutropenia since the age of 10 months. During each episode of the child’s viral infection, absolute neutrophil counts ranged between 0.13 and 0.44 ×10^9^/L, while hemoglobin and platelet values remained within the expected range. Lymphocyte subsets and immunoglobulin levels were normal. Whole-exome sequencing revealed two novel *CXCR2* variants: a frameshift mutation (c.665delT, p.Val222GlyfsTer4) from his mother and a missense mutation (c.748A>C, p.Met250Leu) from his father. He required intermittent courses of granulocyte colony-stimulating factor and broad-spectrum antibiotics during neutropenic fever episodes. This case expands the mutational and phenotypic spectrum of *CXCR2*-related congenital neutropenia and highlights the value of genetic testing for infants with unexplained chronic neutropenia and recurrent infections.

## Introduction

Congenital neutropenia comprises a heterogeneous set of disorders in which circulating neutrophils are persistently reduced, resulting in a variable risk of bacterial and viral infections. Over the past two decades, advances in molecular genetics have led to the identification of several causative genes, including *ELANE*, *GFI1*, *HAX1*, *G6PC3*, *VPS45*, *JAGN1*, *CSF3R*, *SRP54*, *CLPB*, and *WAS* (1). The signaling chemokine receptors plays an essential role in neutrophil homeostasis. CXCR2 facilitates the egress of neutrophils from the bone marrow into the bloodstream, while CXCR4 and its ligand CXCL12 mediate the retention of neutrophils within the bone marrow (2). Disruption of this regulatory balance impairs neutrophil trafficking and leads to neutropenia (2, 3). Biallelic loss-of-function variants in *CXCR2*, although rare, have recently been described as a distinct form of congenital neutropenia (4). These patients typically present in infancy or early childhood with severe neutropenia, recurrent infections, and sometimes oral mucosal lesions. This condition should be distinguished from WHIM syndrome, which is caused by gain-of-function variants in *CXCR4* and is characterized by myelokathexis, recurrent infections, and warts (5). Although both disorders involve abnormalities in chemokine receptor signaling and neutrophil trafficking, their molecular mechanisms and clinical manifestations are distinct.

Here, we present a Chinese infant carrying two novel *CXCR2* variants as compound heterozygotes, thereby broadening the known clinical and genetic spectrum of this entity.

## Case presentation

The patient was a 12-month-old boy, the first child of healthy and non-consanguineous Chinese parents. He was born at full term following an uncomplicated pregnancy and vaginal delivery. There was no family history of recurrent infections, immune deficiencies, or hematologic diseases. His growth and neurodevelopment were appropriate for his age. The patient developed an isolated respiratory infection at 2 months of age, with SARS-CoV-2 RNA detected. The recovery course was uneventful, and no evidence of persistent hematological abnormalities was observed.

Beginning at 10 months of age, he began to experience recurrent respiratory infections every 2–4 weeks, characterized by fever, rhinorrhea, cough, and nasal congestion. During these episodes, severe neutropenia was observed with absolute neutrophil count (ANC) at 0.13–0.44 × 10^9^/L, while levels of hemoglobin and platelets remained stable. Viral pathogens such as rhinovirus and human coronavirus were identified in the patient sample. Empirical intravenous ceftriaxone therapy was initiated in the setting of febrile neutropenia, given the elevated risk of invasive bacterial infection. And intermittent administration of granulocyte colony-stimulating factor (G-CSF) at 3 μg/kg, which reliably increased ANCs. After administration of G-CSF, the white blood cell (WBC) count increased from 5.7 × 10^9^/L to 33.8 × 10^9^/L, and the ANC rose from 0.23 × 10^9^/L to 22.5 × 10^9^/L. Lymphocyte subset analysis revealed preserved immune cell populations without evidence of lymphopenia. Notably, elevated absolute counts of CD3^+^ and CD4^+^ T cells, along with increased expression of activation markers such as CD28 and CD38, suggest an activated immune state (**Tables 1**, **2**).

**Table 1:** The patient’s laboratory findings at diagnosis.

Parameter	Reference range	Laboratory results
WBC	5.5–13.6 × 10^9^/L	6.7
ANC	1.5–5.50 × 10^9^/L	0.13
ALC	2.7–9.10 × 10^9^/L	5.56
HGB	104–143 g/L	116
PLT	191–516 × 10^9^/L	303

WBC, white blood cell; ANC: absolute neutrophil count; ALC, absolute lymphocyte count; HGB, hemoglobin; PLT, platelet.

**Table 2:** Immunologic evaluation at diagnosis.

Parameter	Reference range (/μL)	Result (/μL)
Lymphocyte subsets (absolute counts)		
T cells (CD3^+^)	723–2737	3181 ↑
CD4^+^ T cells	404–1612	2369 ↑
CD4^+^CD28^+^ cells	520–1050	2357 ↑
CD8^+^ T cells	220–1129	685
CD8^+^CD28^+^ cells	190–392	666 ↑
CD8^+^HLA-DR^+^ cells	20–178	45
CD8^+^CD38^+^ cells	157–385	632 ↑
B cells (CD19^+^)	80–616	655 ↑
NK cells (CD3^-^CD56^+^)	84–724	382
Immunoglobulin levels		
IgG	232–1411	960
IgM	0–145	137
IgA	0–83	53

CD, cluster of differentiation; NK, natural killer cells; HLA-DR, human leukocyte antigen–DR isotype; IgG, immunoglobulin G; IgM, immunoglobulin M; IgA, immunoglobulin A.

Whole-exome sequencing was performed using peripheral blood–derived genomic DNA. Exonic regions were enriched using the IDT xGen^®^ Exome Research Panel v1.0 and sequenced on the GenoLab M platform (GenoMind, China) with 150-bp paired-end reads. Raw sequencing data underwent quality control filtering to remove low-quality and adapter-contaminated reads, followed by alignment to the human reference genome (GRCh37/hg19) using Burrows–Wheeler Aligner. Variant calling was performed using SAMtools and bcftools, and functional annotation was conducted using ANNOVAR.

Variants were filtered based on population frequency databases, including 1000 Genomes, ESP6500, and gnomAD, with prioritization of rare protein-altering and splice-site variants consistent with the clinical phenotype and suspected inheritance model. Pathogenicity prediction was assessed using in silico tools including SIFT, PolyPhen-2, MutationTaster, and CADD. Variant interpretation was performed according to the American College of Medical Genetics and Genomics guidelines. Candidate variants were validated by Sanger sequencing.

**Figure 1:**
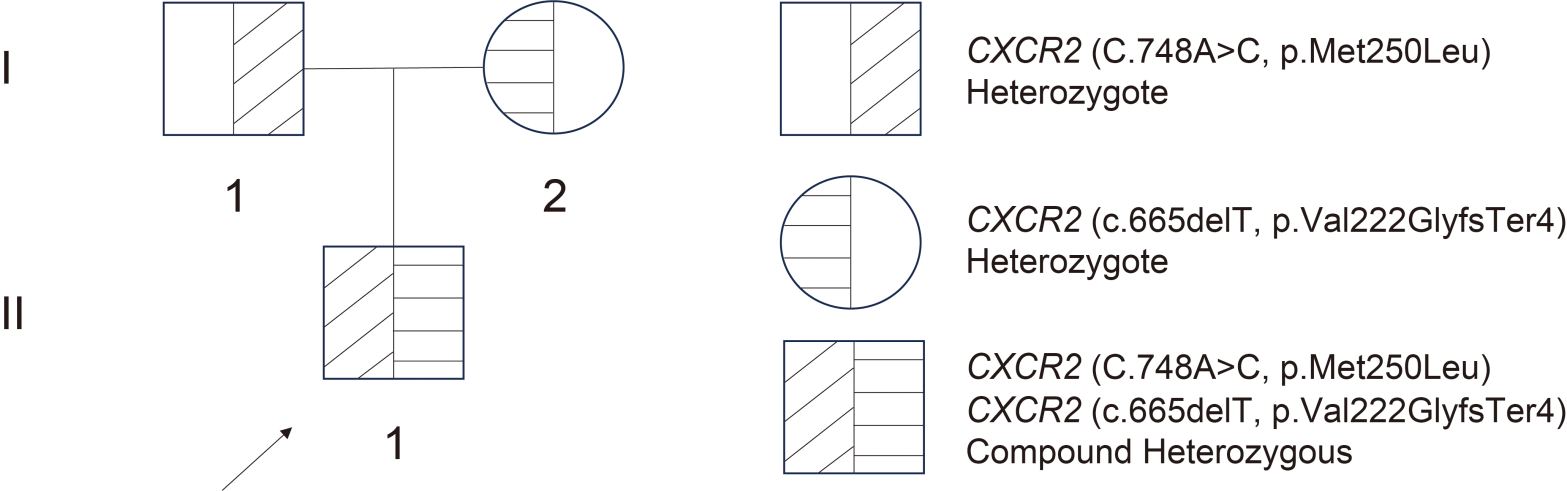
*CXCR2* variants identified in the family. Father:carrying the missense mutation c.748A>C. Mother:carrying the frameshift mutation c.665del. Proband: compound heterozygous mutation.

This analysis identified two heterozygous variants in the *CXCR2* gene (NM_001557.5): a maternally inherited frameshift variant, c.665delT (p.Val222GlyfsTer4), and a paternally inherited missense variant, c.748A>C (p.Met250Leu) (**Figure 1**). **Table 3** summarizes all reported *CXCR2* variants and associated clinical phenotypes. Most cases involve autosomal recessive loss-of-function variants causing chronic neutropenia with recurrent infections, while heterozygous variants may result in milder phenotypes. Our patient demonstrates an infection-triggered intermittent neutropenia, expanding the clinical spectrum of *CXCR2*-related disease.

**Table 3:** Reported *CXCR2* variants and associated clinical phenotypes.

Study	Variant (cDNA, HGVS)	Protein change	Zygosity	Inheritance	Age at onset/diagnosis	Neutropenia phenotype	Main clinical manifestations	Bone marrow findings	G-CSF response
Auer, P.L., et al., 2014 (3)	c.123A>G	p.Lys41Arg	Heterozygous	Inheritance unknown/not established	Adult	Mild neutropenia	Not reported	Not reported	Not required
Auer, P.L., et al., 2014 (3)	c.665C>T	p.Pro222Leu	Heterozygous	Inheritance unknown/not established	Adult	Mild neutropenia	Not reported	Not reported	Not required
Marin-Esteban et al., 2022 (4)	Large deletion (whole CXCR2 gene)	— (loss-of-function)	Homozygous	AR	Childhood	Severe chronic neutropenia	Recurrent gingivitis, oral ulcers	Myelokathexis-like	Responsive
Marin-Esteban et al., 2022 (4)	c.430C>T	p.Arg144Cys	Homozygous	AR	Childhood	Severe chronic neutropenia	Recurrent oral infections	Preserved granulopoiesis	Responsive
Marin-Esteban et al., 2022 (4)	c.635C>T	p.Arg212Trp	Homozygous	AR	Childhood	Chronic neutropenia	Gingivitis, oral ulcers	No maturation arrest	Responsive
Marin-Esteban et al., 2022 (4)	c.635C>T + c.866C>T	p.Arg212Trp + p.Arg289Cys	Compound heterozygous	AR	Childhood	Chronic neutropenia	Mucosal infections	Preserved granulopoiesis	Responsive
Klimiankou et al., 2024 (11)	c.431G>A	p.Arg144His	Homozygous	AR	Adult (~30 y)	Severe chronic neutropenia	Respiratory infections, skin infections	Hypercellular marrow, myeloid expansion	Responsive
Klimiankou et al., 2024 (11)	c.431G>A + c.720_727del	p.Arg144His + p.Lys240Asnfs*45	Compound heterozygous	AR	Adolescence (~14 y)	Severe chronic neutropenia	Pharyngitis, bronchitis, oral ulcers	Increased granulopoiesis, no arrest	Responsive
Klimiankou et al., 2024 (11)	c.431G>A	p.Arg144His	Heterozygous	AD-like (incomplete penetrance)	Adult (~32 y)	Mild neutropenia	Asymptomatic	Not available	Not required
Present case	c.665delT + c.748A>C	p.Val222Glyfs*4 + p.Met250Leu	Compound heterozygous	AR	Infancy (10 months)	Intermittent severe neutropenia (infection-triggered)	Recurrent febrile respiratory infections	Not performed	Responsive

AR, autosomal recessive; AD-like, autosomal dominant-like; HGVS, Human Genome Variation Society nomenclature; G-CSF, granulocyte colony-stimulating factor.

At the last follow-up in June 2025, the patient continued to exhibit intermittent neutropenia, particularly during infectious episodes. Administration of G-CSF resulted in a rapid increase in neutrophil counts and clinical improvement. To date, no severe complications or disease progression have been observed.

## Discussion

The processes of neutrophil production, release, and clearance are tightly controlled and involve several chemokine receptors (1, 2). Among these, the chemokine receptor *CXCR2* plays a central role in mediating neutrophil trafficking through its interaction with ELR+ CXC chemokines such as CXCL8 (IL-8) (6, 7). Although both *CXCR2* deficiency and WHIM syndrome affect neutrophil trafficking through chemokine receptor dysregulation, they represent distinct clinical entities. WHIM syndrome results from gain-of-function *CXCR4* variants and is typically associated with myelokathexis and warts, whereas *CXCR2* deficiency is caused by loss-of-function variants that primarily impair neutrophil mobilization (4, 5). *CXCR2* signaling is critical not only for neutrophil chemotaxis toward inflammatory sites but also for their mobilization from the bone marrow into peripheral circulation (2). Disruption of this pathway has been shown to impair neutrophil recruitment and host defense, predisposing affected individuals to recurrent infections (8, 9).

In this case, we describe an infant with recurrent respiratory infections and intermittent severe neutropenia who harbors compound heterozygous variants in *CXCR2*, including a maternally inherited frameshift variant (c.665delT, p.Val222GlyfsTer4) and a paternally inherited missense variant (c.748A>C, p.Met250Leu). The frameshift variant is predicted to result in a truncated protein and likely represents a loss-of-function allele, whereas the missense variant may affect receptor structure or downstream signaling (10). Data from flow-cytometric studies suggest a dosage effect, with heterozygous carriers showing intermediate *CXCR2* expression compared with patients and controls (4). Klimiankou et al. further demonstrated that *CXCR2* surface expression levels correlate with neutrophil release efficiency, reinforcing this model (11).

**Table 3** summarizes all reported *CXCR2* variants and associated clinical phenotypes, highlighting shared and distinct features across cases. Notably, our patient exhibited an infection-triggered intermittent neutropenia, which contrasts with the persistent neutropenia typically observed in previously reported cases (3, 4, 11). In addition, flow cytometry revealed increased CD3^+^T cells and related T-cell subsets, a finding not prominently reported in prior *CXCR2*-deficient patients. Possible explanations include reactive adaptive immune activation secondary to recurrent infections, compensatory expansion of adaptive immune cells due to impaired neutrophil-mediated innate immunity, and age-related immunological differences.

The phenotype of our patient was characterized by intermittent severe neutropenia, recurrent infections, preserved hemoglobin and platelet counts, and otherwise normal immune parameters. Most reported patients exhibit early onset neutropenia and recurrent infections, sometimes with oral lesions, but without syndromic manifestations typical of WHIM syndrome (4, 5). Bone marrow evaluation in such cases often demonstrates intact granulopoiesis, occasionally revealing myelopathies. Reported variants to date include missense mutations (e.g., p. Arg144Cys, p. Arg212Trp, p. Arg289Cys) and deletions that impair receptor signaling (4). Our case demonstrates that *CXCR2* deficiency can manifest as stress- or infection-induced intermittent neutropenia.

Functional validation of the identified *CXCR2* variants, including neutrophil chemotaxis assays and *CXCR2* surface expression studies, was not performed due to limited sample availability and the patient’s young age. Such analyses would have provided direct evidence for the pathogenicity of the variants, particularly the missense mutation, and future studies are warranted to address this limitation. Bone marrow evaluation was not performed, which may have provided important insights into granulocyte maturation defects or other underlying marrow pathology.

Current evidence regarding *CXCR2* loss-of-function is largely derived from animal models and a limited number of human cases (2, 8, 12). These studies consistently demonstrate impaired neutrophil mobilization, increased susceptibility to infections, and abnormal neutrophil retention within the bone marrow. In contrast to the persistent neutropenia typically reported, our patient exhibited infection-triggered intermittent severe neutropenia, suggesting that *CXCR2*-related disorders may present with a broader phenotypic spectrum.

The episodic nature of neutropenia may be explained by the role of *CXCR2* in neutrophil mobilization during inflammatory responses. Under physiological conditions, chemokines activate *CXCR2* to promote neutrophil egress from the bone marrow and recruitment to sites of infection (6). In the presence of CXCR2 dysfunction, this process is impaired, leading to transient depletion of circulating neutrophils during periods of increased demand (6, 12). Defective chemotaxis may also result in ineffective pathogen clearance, amplifying inflammatory signals and contributing to recurrent infections (13).

Experimental studies further support the role of *CXCR2* in neutrophil mobilization. *CXCR2* knockout mice demonstrate abnormal neutrophil retention within the bone marrow and impaired neutrophil trafficking (2). However, the extrapolation of findings from murine models to human disease should be interpreted cautiously, given potential interspecies differences in neutrophil biology and chemokine signaling pathways.

Despite these defects, patients carrying compound heterozygous *CXCR2* variants may retain partial responsiveness to G-CSF, although the required dose and magnitude of response appear variable. Marin-Esteban et al. reported effective hematologic responses at doses ranging from 2 to 5 μg/kg (4). Similarly, our patient consistently exhibited hematologic recovery at a dose of 3 μg/kg during episodes of severe neutropenia.

Importantly, this patient demonstrated a robust response to G-CSF, with a marked increase in absolute neutrophil count following administration. G-CSF is known to stimulate granulopoiesis and enhance neutrophil release from the bone marrow, potentially bypassing, at least in part, the defective *CXCR2*-mediated signaling pathway (14). This observation is consistent with prior reports suggesting that G-CSF can be effective in certain forms of congenital or functional neutropenia (15). Our findings further support the use of G-CSF as a therapeutic strategy in patients with *CXCR2*-related neutrophil disorders, although the optimal dosing and long-term outcomes remain to be established.

Our results further support the use of G-CSF as a therapeutic strategy for patients with *CXCR2*-related neutrophil dysfunction. It is noteworthy that the risk of G-CSF-induced treatment in severe congenital neutropenia with MDS/AML has been extensively documented in the literature. Therefore, the optimal dosage, long-term efficacy, and safety profile of G-CSF as a therapeutic approach for *CXCR2*-related neutrophil dysfunction remain to be further elucidated.

This case expands the current understanding of *CXCR2*-associated disease in several important ways. First, it identifies a novel combination of compound heterozygous variants in *CXCR2*, thereby enriching the mutational spectrum of this gene. Second, it highlights a previously underrecognized clinical phenotype characterized by infection-associated, intermittent severe neutropenia. Third, it provides clinical evidence supporting the efficacy of G-CSF in this context. Together, these findings underscore the importance of considering *CXCR2* variants in the differential diagnosis of unexplained recurrent infections with fluctuating neutrophil counts, particularly when standard immunological evaluations are unremarkable.

Several limitations should be acknowledged. Bone marrow evaluation and functional validation studies, including neutrophil chemotaxis and *CXCR2* expression analyses, were not performed. In addition, segregation analysis was restricted to the nuclear family, and long-term follow-up was relatively short, precluding assessment of natural disease progression or late-onset complications associated with prolonged G-CSF therapy. Future studies incorporating functional assays, bone marrow assessment, extended segregation analysis, and longer follow-up will be essential to further define genotype–phenotype correlations in *CXCR2*-related neutropenia.

In conclusion, we report a rare case of *CXCR2* compound heterozygous variants associated with recurrent respiratory infections and intermittent severe neutropenia in infancy. This case broadens the phenotypic and genotypic spectrum of *CXCR2*-related disorders and provides further evidence for the clinical utility of G-CSF in managing this condition.

## Conclusion

We report a novel compound heterozygous *CXCR2* variant in an infant with neutropenia. This case expands both the genetic and clinical spectrum of *CXCR2*-related disease. Importantly, it highlights an intermittent, infection-triggered neutropenia phenotype, underscoring the need to consider *CXCR2* deficiency even in atypical presentations.

## Data availability statment

The datasets presented in this article are not readily available because of ethical and privacy restrictions. Requests to access the datasets should be directed to the corresponding authors.

## References

[1] Parisi X Bledsoe JR . Discerning clinicopathological features of congenital neutropenia syndromes: an approach to diagnostically challenging differential diagnoses. J Clin Pathol. (2024) 77:586–604. doi: 10.1136/jcp-2022-208686. PMID: 38589208

[2] Eash KJ Greenbaum AM Gopalan PK Link DC . CXCR2 and CXCR4 antagonistically regulate neutrophil trafficking from murine bone marrow. J Clin Invest. (2010) 120:2423–31. doi: 10.1172/jci41649. PMID: 20516641 PMC2898597

[3] Auer PL Teumer A Schick U O'Shaughnessy A Lo KS Chami N . Rare and low-frequency coding variants in CXCR2 and other genes are associated with hematological traits. Nat Genet. (2014) 46:629–34. doi: 10.1038/ng.2962. PMID: 24777453 PMC4050975

[4] Marin-Esteban V Youn J Beaupain B Jaracz-Ros A Barlogis V Fenneteau O . Biallelic CXCR2 loss-of-function mutations define a distinct congenital neutropenia entity. Haematologica. (2022) 107:765–9. doi: 10.3324/haematol.2021.279254. PMID: 34854278 PMC8883555

[5] Kawai T Malech HL . WHIM syndrome: congenital immune deficiency disease. Curr Opin Hematol. (2009) 16:20–6. doi: 10.1097/moh.0b013e32831ac557. PMID: 19057201 PMC2673024

[6] Rajarathnam K Schnoor M Richardson RM Rajagopal S . How do chemokines navigate neutrophils to the target site: dissecting the structural mechanisms and signaling pathways. Cell Signal. (2019) 54:69–80. doi: 10.1016/j.cellsig.2018.11.004. PMID: 30465827 PMC6664297

[7] Matsushima K Yang D Oppenheim JJ . Interleukin-8: an evolving chemokine. Cytokine. (2022) 153:155828. doi: 10.1016/j.cyto.2022.155828. PMID: 35247648

[8] Nguyen CH Zmajkovicova K Sekirnik A Taplin S Defontis M Bledsoe JR . CXCR4 antagonism corrects neutrophil abnormalities and reduces pneumonia severity in a pharmacological mouse model of CXCR2 loss-of-function-mediated neutropenia. Front Immunol. (2025) 16:1658987. doi: 10.3389/fimmu.2025.1658987. PMID: 41451234 PMC12727938

[9] Herbold W Maus R Hahn I Ding N Srivastava M Christman JW . Importance of CXC chemokine receptor 2 in alveolar neutrophil and exudate macrophage recruitment in response to pneumococcal lung infection. Infect Immun. (2010) 78:2620–30. doi: 10.1055/s-0030-1251250. PMID: 20368349 PMC2876546

[10] Semsarian C Ingles J Barratt Ross S Dunwoodie SL Bagnall RD Kovacic JC . Precision medicine in cardiovascular disease: genetics and impact on phenotypes: JACC Focus Seminar 1/5. J Am Coll Cardiol. (2021) 77:2517–30. doi: 10.1007/978-1-59259-878-6_1. PMID: 34016265

[11] Klimiankou M Tesakov I Tsaknakis G Boutakoglou E Mavroudi I Ritter M . Expanding the genetic landscape of congenital neutropenia: CXCR2 mutations in three families revealed through whole exome sequencing. Haematologica. (2024) 109:4140–4. doi: 10.3324/haematol.2024.285569. PMID: 39086298 PMC11609805

[12] Delobel P Ginter B Rubio E Balabanian K Lazennec G . CXCR2 intrinsically drives the maturation and function of neutrophils in mice. Front Immunol. (2022) 13:1005551. doi: 10.3389/fimmu.2022.1005551. PMID: 36311783 PMC9606682

[13] Dinauer MC . Inflammatory consequences of inherited disorders affecting neutrophil function. Blood. (2019) 133:2130–9. doi: 10.1182/blood-2018-11-844563. PMID: 30898864 PMC6524563

[14] Kaushansky K . Lineage-specific hematopoietic growth factors. N Engl J Med. (2006) 354:2034–45. doi: 10.1056/nejmra052706. PMID: 16687716

[15] Mehta HM Malandra M Corey SJ . G-CSF and GM-CSF in neutropenia. J Immunol. (2015) 195:1341–9. doi: 10.4049/jimmunol.1500861. PMID: 26254266 PMC4741374

